# Potential Sources and Roles of Adaptive Immunity in Age-Related Macular Degeneration: Shall We Rename AMD into Autoimmune Macular Disease?

**DOI:** 10.1155/2014/532487

**Published:** 2014-04-30

**Authors:** Serge Camelo

**Affiliations:** Private Practice, 193 avenue du Président Wilson, La Plaine, 93210 Saint-Denis, France

## Abstract

Age-related macular degeneration (AMD) is the leading cause of vision loss in the elderly throughout the industrialized world. Its most prominent pathologic features are lesions involving the retinal pigment epithelium (RPE) the Bruch's membrane, the degeneration of photoreceptors, and, in the most aggressive cases, choroidal neovascularization. Genetic associations between the risk of developing AMD and polymorphism within components of the complement system, as well as chemokine receptors expressed on microglial cells and macrophages, have linked retinal degeneration and choroidal neovascularization to innate immunity (inflammation). In addition to inflammation, players of the adaptive immunity including cytokines, chemokines, antibodies, and T cells have been detected in animal models of AMD and in patients suffering from this pathology. These observations suggest that adaptive immunity might play a role in different processes associated with AMD such as RPE atrophy, neovascularization, and retinal degeneration. To this date however, the exact roles (if any) of autoantibodies and T cells in AMD remain unknown. In this review we discuss the potential effects of adaptive immune responses in AMD pathogenesis.

## 1. Introduction

Age-related macular degeneration (AMD) is the leading cause of irreversible blindness in the industrialized world [1]. There are two clinical forms of late AMD: the fast developing exudative form (wet AMD) defined by choroidal neovascularization (CNV) and the more slowly developing atrophic form known as geographic atrophy (GA or dry AMD). Wet AMD is characterized by subretinal extravasations of neovessels and hemorrhage under and into the photoreceptor cell layer which induces photoreceptor cell loss [2]. Dry AMD is characterized by retinal pigment epithelium (RPE) atrophy and photoreceptor degeneration [2].

Both wet AMD and dry AMD are complex multifactorial events. Aging [3, 4] and environmental factors such as smoking history [5], oxidative stress [6–9], and more recently low grade inflammation [8, 10, 11] are clearly involved in both CNV and dry AMD pathogenesis. First evidence that innate immunity was at play during AMD came from genetic studies showing that people with polymorphisms in the complement factor H (CfH) [12–15] had an increased prevalence of AMD. Since then, polymorphisms in C3 [16, 17] and complement factors B (CfB) [17, 18] and I (CfI) [19] have been linked to AMD. Moreover, polymorphisms in the CX3CR1 gene, which, in the eye, is specifically expressed on microglia, have also been associated with AMD [20–22]. The exact role of polymorphisms in the CX3CR1 gene during AMD remains however unknown [23]. In CX3CR1 deficient mice, accumulation of microglia and macrophages (MΦ) in the subretinal space has been observed. Similarly, in humans it has been proposed that mutations in the CX3CR1 gene would induce recruitment of monocytes/microglia into the subretinal space in the eyes of patients with AMD [22, 24–26]. It has also been shown that, in mice deficient in the CX3CR1 gene, phagocytosis of cellular debris and lipids by monocytes in the subretinal space [22, 24–26] is important in drusen formation and photoreceptors degeneration. Finally in mouse models of CNV the release of VEGF by monocytes recruited to the subretinal space plays a crucial role in choroidal blood vessel growth [22, 27–29]. Therefore CX3CR1-dependent regulation of monocytes/ MΦ recruitment in the subretinal space appears to be involved in the development of both wet and dry AMD [22, 26–30]. The role of innate immunity in AMD has been further demonstrated by the elevated plasma levels of activated complement factor 3 (C3a) [31] and C-reactive protein (CRP), a marker of inflammation [32, 33]. Recently, human and animal studies indicated that adaptive immunity directed towards the retina and/or the RPE is also involved in wet and dry AMD [34–37]. In this review we will discuss the potential sources and roles of adaptive immune responses in the various processes leading to the exudative and atrophic forms of AMD.

## 2. Role of Antiretinal and Anti-RPE Autoantibodies Found in the Serum and Drusen of AMD Patients

As early as 1990, autoantibodies to retinal astrocytes were detected in serum of patients with AMD [38], suggesting that antiretinal autoantibodies could play a role in this disease. Accordingly, using indirect immunohistochemistry, Patel and colleagues showed that serum from people with age-related maculopathy (ARM) exhibited higher levels of retinal autoantibodies than serum of controls. These autoantibodies were specific for all layers of the retina [39]. Autoantibodies present in AMD patient's serum have been mostly associated with the exudative form of AMD. Multiple retinal autoantigens have been described. These antiretinal antibodies from AMD patients partly react with unknown retinal proteins of varying molecular weight [40] including a 68 kDa neurofilament protein found in photoreceptors outer segments [41]. A more recent study showed that antiretinal antibodies recognizing glial fibrillary protein (GFAP, expressed by Müller cells and astrocytes in the retina) and *α*-enolase were found at significantly higher level in AMD patient's blood than in normal controls and in patients with other retinal diseases [42]. Using an antigen microarray technique Mohoroshi and colleagues revealed the association of both forms of AMD with elevated serum level of specific autoantibodies against no less than 31 antigens [43, 44]. Among IgG and IgM antiretinal autoantibodies found at elevated levels in the serum of patients with AMD, those specific for phosphatidyl serine (PS), JO-1, U1-snRNP-68, elastin, cytochrom C, PM/scl-100, and collagen III were associated specifically with CNV but not with dry AMD [43, 44]. In contrast, IgG and IgM recognizing fibronectins were uniquely associated with dry AMD. In this study, these authors also showed that increased levels of autoantibodies recognizing constituents of drusen and extracellular matrix in Bruch's membrane were also present in AMD patients [43, 44]. However, in another study, there was no association between AMD development and antiretinal autoantibodies with various retinal antigen specificities [45]. Absence of correlation between autoantibodies and AMD, in this study however, could be due to the limited sample size. Nevertheless, taken collectively the numerous studies demonstrating the presence of autoantibodies recognizing retinal and RPE proteins in sera of AMD patients strongly suggest that they play a role in AMD.

In addition to specific retinal and RPE proteins, adjuncts generated by free radical-induced oxidation are also recognized by autoantibodies [46]. Oxidation of docosahexaenoate- (DHA-) containing lipids generates carboxyethylpyrrole (CEP) protein adducts. Immunocytochemistry localized CEP to the outer segments of photoreceptor rod and the RPE in mouse retina and demonstrated more intense CEP immune-reactivity in photoreceptors from a human AMD donor compared with healthy human retina. It was also shown that these CEP adducted-proteins are more abundant in drusen [47] and blood from AMD patients than in normal human donors [46, 48]. Moreover, sera from AMD patients demonstrated higher mean titers of anti-CEP autoantibody than controls [46]. Among individuals exhibiting both CEP-antigen and specific autoantibody levels above the means for non-AMD controls, 92% had AMD. Importantly, in mice, immunization with CEP-adducted mouse serum albumin leads to the appearance of antibodies specific to CEP and induced RPE lesions and photoreceptor degeneration [6, 7]. These results strongly suggest that autoantibodies specifically recognizing CEP-adducted proteins are linked to AMD susceptibility.

Further studies looking for a specific autoantibody signature of AMD (for which anti-PS and/or anti-CEP are clear candidates) must be undertaken before we can use it as a biomarker for this disease. This remains a complicated task due to two main reasons: (i) the great variety of targets recognized by autoantibodies of multiple Ig subtypes present in the serum of AMD patients and (ii) to a certain extent, the identical specificities of antiretinal autoantibodies found in sera of AMD patients, in sera of controls, and in sera of patients suffering from other ocular and nonocular diseases [49]. For instance antibodies against *α*-enolase have been associated not only with AMD [42] but also with cancer associated retinopathy (CAR) and nonparaneoplastic autoimmune retinopathy (npAIR) [35, 50, 51]. Similarly, antibodies specific for *α*B-crystallin, which is the basic structural component of multiple heat chock proteins (HSP), are found not only in sera of AMD patients [42] but also in sera of patients with uveitis [52]. Moreover, *α*-crystallin is also recognized by autoantibodies during multiple sclerosis [53] and cardiovascular diseases [54].

At that point we still do not know whether autoantibodies are specifically part of AMD processes and could be one of the causes of the disease or if they are just a signature of other factors involved in AMD pathology, for instance, ageing. Indeed, it is known that autoantibodies levels increase and tolerance mechanisms fail with age in a phenomenon called “immunosenescence” [55, 56]. Alternatively autoantibodies specific of retinal and/or RPE antigens could be induced after ocular tissues damage occurring during AMD. Supporting this hypothesis, it has been reported that laser treatment in rabbits' eyes releases ocular antigens in the circulation and that it generates the production of antiretinal antibodies observed 3 months later in their serum [57]. Another possibility is that antiretinal and RPE autoantibodies are the footprint of an etiologic agent associated with AMD. This possibility will be discussed in the last chapter of this review.

**Figure 1:**
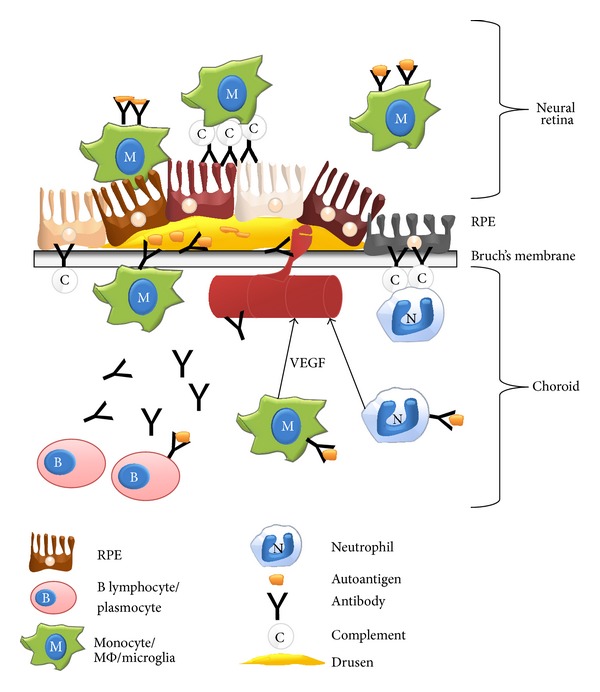
Potential mechanisms of action of autoantibodies in AMD. Classical activation of complement pathway by autoantibodies produced by autoreactive plasmocytes could destroy directly RPE. In addition, autoantibodies could recruit, activate, and induce RPE phagocytosis by macrophages and neutrophils in a complement dependent or independent manner. Moreover, autoantibodies could induce choroidal vessels involution by inhibiting vessel growth or in contrary enhancing choroidal neovascularization by inducing proangiogenic factors production by macrophages and neutrophils. VEGF: vascular endothelial growth factor; RPE: retinal pigmented epithelium; location of the neural retina is mentioned but not represented.

Independently of knowing whether antiretinal and RPE autoantibodies preexist to AMD or are produced in reaction to ocular tissues damage after AMD starts, autoantibodies have the capacity to participate in AMD evolution. A group has been extensively studying the role of antiretinal autoantibodies in several models of retinal degeneration. They have shown that autoantibodies against recoverin killed photoreceptors* in vivo* [58]. Moreover they have demonstrated that adoptive transfer of antiretinal autoantibodies obtained from Royal College of Surgeons' (RCS) rats (a model of inherited retinal degeneration) induced disruption of the blood retinal barrier (BRB), upregulated CCL2, attracted macrophages in the retina, and increased the level of photoreceptors apoptosis [59]. Accordingly, very recently it has been shown in WT and* C1q* ^−/−^ mice that immune complex (IgG linked to antigens) deposition in the retina led to a robust inflammatory response with activation of microglia and recruitment of myeloid cells. These studies suggest that immune complexes may contribute to several phenomena observed during AMD pathogenesis through interaction of IgG with Fc*γ*Rs [60]. Inflammation and photoreceptors degeneration are observed during the course of AMD especially the dry form of the disease. Interestingly, Mohoroshi and coworkers also reported that antibodies from patients with AMD (particularly those with CNV) augmented significantly the tube formation of endothelial cells cultivated on matrigel* in vitro*, indicating a potential role of autoantibodies in “wet” AMD also [44]. In conclusion, further studies are required to definitely link (or not) the presence of antiretinal and RPE autoantibodies to AMD development (the potential effects of antibodies during AMD are summarized in Figure 1).

## 3. Presence and Role of T Lymphocytes in the Eyes of AMD Patients

**Figure 2:**
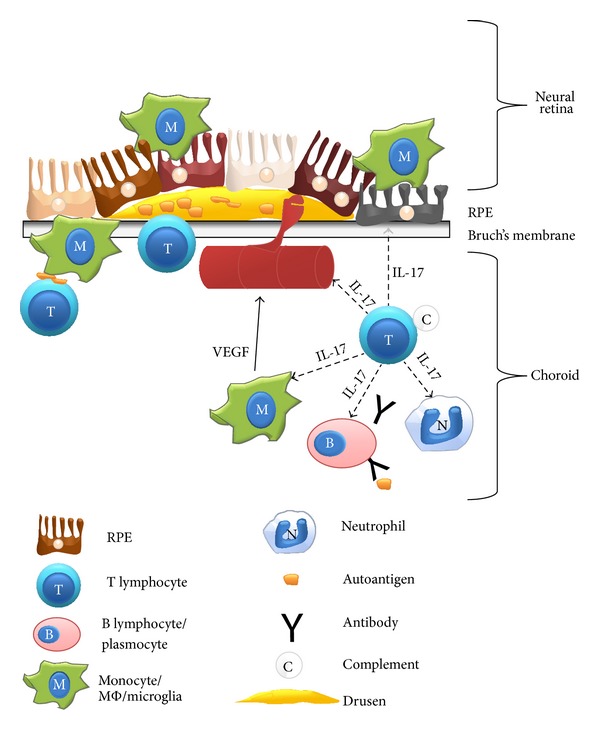
Potential mechanisms of action of autoreactive T cells and IL-17 in AMD. Antigen presentation by macrophages to autoreactive T cells could lead to alterations or the destruction of Bruch's membrane and RPE by classical FAS-FASL interaction or granzyme/perforin production. Autoreactive T cells can also participate in AMD development via cytokines production. For instance, in the presence of complement proteins, T cells may produce IL-17. IL-17 is toxic to photoreceptors and RPE cells. IL-17 could also induce macrophages and neutrophils activation that can destroy RPE or produce proangiogenic factors (VEGF). In addition, IL-17 has been reported to be a proangiogenic molecule itself and can participate in choroidal neovascularization. Finally IL-17 and other cytokines produced by activated autoreactive T lymphocytes may induce the production of antibodies specific to RPE and retinal proteins. VEGF: vascular endothelial growth factor; RPE; retinal pigmented epithelium; location of the neural retina is mentioned but not represented.

Similar to the presence of autoantibodies in AMD, T cells accumulation has been observed in AMD ocular tissues and blood samples. Almost 30 years ago, Penfold and colleagues detected by electron and light microscopy the presence of lymphocytes in the eyes of AMD patients. From these seminal observations, they proposed that lymphocytes may play a role in neovascularization, atrophy of the RPE, and breakdown of Bruch's membrane at early and late stages of macular degeneration [61, 62] (Figure 2). More recently, CD8^+^ T cells have been observed by fluorescence microscopy in the choroid of frozen sections of eyes from AMD patients [63]. Gene and protein expression of the chemokines CXCL10 and CXCL11, both chemotactic for CXCR3^+^ T cells, are augmented in ocular fluids of AMD patients [64, 65]. Antigen presentation to potential recruited T cells in the eye is possible since MHC class II expression has been observed on RPE [66] and is enhanced on microglia during aging and AMD [5, 24]. In aging mice, at the mRNA level, several genes specific for T cells (CD3, CD8, T cell receptor, and LY75), antigen presentation (*β*2-microglobulin and H2 molecules), T lymphocytes chemotaxis (CXCL9, CXCL10, CXCL11, and CCL5), and adhesion of leucocytes (ICAM-1) were overexpressed in the RPE/choroid complex and the retina [67, 68]. In murine models of light induced retinal degeneration, mild T cells infiltration [69] as well as increased levels of dendritic cells (DC) expressing MHC class II in the retina has also been observed [70]. All these reports suggest a potential role for adaptive immunity, and in particular for T lymphocytes, in the pathogenesis of AMD.

For many years, the pathologic or protective role of recruited T lymphocytes during AMD remained a subject of debate. In 2005 however, it was shown that polymorphisms in MHC classes I and II molecules influenced the development of AMD, suggesting that T cells response might indeed play a role in AMD pathology [71]. This hypothesis has been confirmed very recently by the study from Faber et al., who reported elevated percentages of CD56^+^ and CD28^−^ memory T cells in the blood of AMD patients in comparison to non-AMD controls. Higher levels of CD56^+^ and CD28^−^ memory T cells increase 3.5 times the risk of developing AMD [72]. The risk of AMD is further augmented by 13.3 times in the case of persons with enhanced blood levels of memory T cells and exhibiting at least one CFH H402 risk allele [72]. This study indicates a direct cooperative effect of both risk factors on AMD outcome and it is the clear demonstration that T cells contribute importantly to AMD pathogenesis.

So far, there is no direct study of the effects of T lymphocytes on ocular tissues during AMD. However, many lessons can be drawn from the observations reported from the studies of experimental autoimmune uveitis (EAU) [73]. EAU in animals serves as a model of human posterior autoimmune uveitis. EAU can be induced in many rodents either by immunization with retinal antigens (interphotoreceptor retinoid-binding protein (IRBP) in mice or S-Antigen in rats) or by adoptive transfer of uveitogenic T cells lines [74]. T lymphocytes are essential to induce retinal destructions both in humans and in animals. T cells are deleterious via cell death inducing molecules (FAS-FASL interaction, granzyme/perforin production) but mostly by secreting cytokines in the retinal tissue [73]. Several years ago the prototypical Th-1 cytokine IFN-*γ* was considered the most important to explain retinal destruction during uveitis [74]. However, recently it has been shown that T lymphocytes producing IL-17 are particularly involved in uveitis [73, 75]. It is therefore important to study the potential effects of IL-17 during AMD to understand the role of T cells in this pathology.

## 4. Potential Roles of IL-17 in AMD

Several subsets of T lymphocytes can be distinguished based on the cytokines they produce. Because IL-17 has been previously involved in many inflammatory and autoimmune diseases including autoimmune uveitis [75–79], a particular interest has been raised on CD3^+^ T cells, either Th (CD4^+^) or Tc (CD8^+^) that produce IL-17, during AMD pathogenesis [80]. It should be kept in mind that IL-17 can also be secreted by several other cell types such as *γδ*T cells, NKT cells, MΦ, and innate lymphoid cells (ILC) [76–79]. Conventional T cells producing IL-17 should be seen integrated in a global “autoimmune” process involving both innate and adaptive immune components and disrupting the normal retinal homeostasis, consequently causing AMD.

In support of the potential role of IL-17 during AMD, increased levels of IL-17 have been detected in the serum of AMD patients compared to IL-17 levels in sera of age-matched controls [81]. The presence of IL-17, reported in ocular tissues of AMD patients [82], suggests that IL-17-producing cells are involved in several pathological processes observed in AMD. In addition, Hasegawa et al. have shown that IL-17 produced by *γδ*T and ILC promotes experimental intraocular neovascularization in laser induced CNV in mouse [83]. The role of IL-17 during AMD has been definitely proven by studying the methylation level of the IL-17RC receptor in monocytes isolated from groups of twins in which one sibling presented signs of AMD but not the other one [84]. DNA methylation regulates gene expression pattern depending on environmental stimuli. Indeed, DNA methylation mediates silencing of gene expression via modifications of the chromatin structure [85]. Thus, hypomethylated genes are prone to be more “expressed” than methylated ones. Lai Wei et al. showed that the frequency of CD14^+^ monocytes expressing the IL-17RC receptor was superior in the blood of siblings with AMD than in their respective sibling without AMD and used as normal controls. They reported that methylation level of the IL-17RC receptor in these CD14^+^ monocytes was significantly reduced in siblings with AMD in comparison to their respective healthy siblings, indicating that the IL-17RC receptor expression is induced during AMD [84]. In addition, the IL-17RC receptor was more expressed in the macula of patients with the dry or the wet form of AMD than in non-AMD controls macula, at the mRNA and proteins levels [84]. These data correlations are strong enough to suggest that IL-17 interaction with its IL-17RC receptor expressed on CD14^+^ monocytes in the blood and in the macula could play a role in AMD.

IL-17 produced by T cells (and other cells types) would be involved in AMD evolution via several mechanisms (summarized in Figure 2). For instance, IL-17 has been shown to be able to recruit monocytes [86, 87], to activate MΦ [88], and to increase MΦ phagocytic capacity [89, 90]. Thus, IL-17 potentially enhances the pathologic roles of MΦ during AMD. Similar effects of IL-17 have been reported on neutrophils [91]. IL-17 is also neurotoxic [92–94] and thus could participate in photoreceptors destruction. Moreover, in experimental autoimmune encephalitis, a model of multiple sclerosis, it has been observed that IL-17 is able to breach tight junction of the blood-brain barrier* in vivo* [92]. Accordingly, destruction of RPE monolayer has been achieved by high concentrations of IL-17* in vitro* [95].

In summary, IL-17 could be a key player during all forms of AMD and may even convert one form into the other. Indeed, IL-17 can induce the destruction of photoreceptors and of the RPE layer which is observed during the dry form of AMD. IL-17 could also be involved in the wet form of AMD since IL-17 has been shown to participate in vessel growth in the subretinal space during CNV in the mouse [83]. IL-17 is therefore a good candidate to promote CNV during the exudative form of AMD either directly, by enhancing the growth of endothelial cells in the presence of angiogenic factors [96], and/or indirectly, by inducing the production of VEGF by other cells types [97].

## 5. What Is the Source of Antiretinal T Cells and Antibodies?

**Figure 3:**
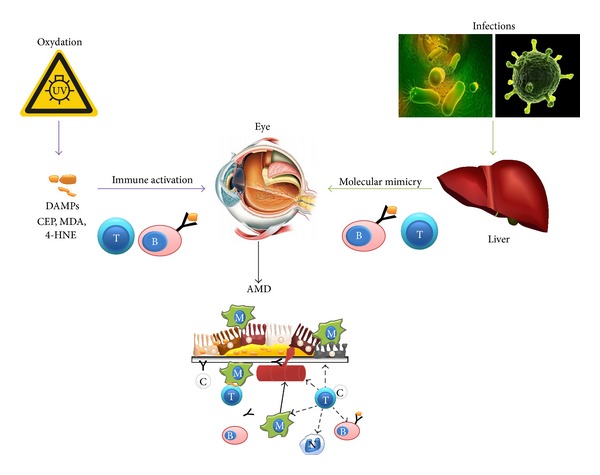
Potential sources of autoimmunity in AMD. Activation of autoreactive B and T lymphocytes could result from two distinct pathways (see text). On the one hand, oxidative stress could induce the release of danger associated molecular patterns (DAMPs, such as CEP, MDA, or 4-HNE). These DAMPs will activate specific lymphocytes. Since DAMPs are found in large amount in ocular tissues, an immune response against RPE and retinal tissues could develop. On the other hand, total pathogen burden, that is, lifetime infections (of the liver, e.g.) by bacteria and viruses, would induce activation of infectious agents specific T and B lymphocytes. Because of the putative similarities between pathogen's and eye's antigens these activated T and B lymphocytes could recognize self-antigens of the eye leading to alterations of the RPE and retinal tissues observed during AMD. Symbols representing T and B lymphocytes, monocytes/MΦ, neutrophils, antibodies, and antigens are identical to those used in Figures 1 and 2. RPE: retinal pigmented epithelium.

Two different mechanisms at least could explain the presence of an autoimmune reaction in AMD: (i) oxidative stress and (ii) infection of the host by pathogens (Figure 3).

Oxidative stress has been recognized as one of the major risk factors in the development of AMD [6–9]. The link between oxidative stress and autoimmune responses could happen as follows. The immune system can be alarmed by evolutionary conserved endogenous structures termed damage-associated molecular patterns (DAMPs) [98]. Oxidative stress is a common source of tissue and cellular injury and is also a potential source of DAMPs [99, 100]. Oxidative damage, mediated by reactive oxygen species, generates deleterious reactive aldehydic byproducts including carboxyethylpyrrole (CEP), malondialdehyde (MDA), and 4-hydroxynonenal (4-HNE) and advanced glycation end (AGE) products such as carboxymethyllysine (CML) and pentosidine [99]. Proteins modified with aldehydic adducts (MDA, 4-HNE) or CEP adducts become more immunogenic [6, 99–101]. For example, as already mentioned, mice immunized with the CEP-adducted proteins develop cardinal features of AMD [6]. The aged macula, as well as drusen, a hallmark of AMD, accumulates a number of oxidation chemical species including MDA [102], pentosidine [103], CML [48, 104], and CEP [46, 47]. Therefore, oxidation of lipids and proteins could induce the generation of new autoantigens to which the immune system has not been “tolerized” [105]. Autoreactive T cells recognizing these neoantigens, as DAMPs, could provide help to autoreactive B cells that will secrete autoantibodies recognizing a large spectrum of retinal and RPE antigens modified by oxidation byproducts [99]. Oxidative stress could thus initiate an autoimmune type of response leading to AMD (Figure 3).

Alternatively, autoantibodies and autoimmune T cells observed during AMD could have been generated following exposure to pathogens, for instance, during infections with viruses or bacteria (Figure 3). It has been previously reported that an autoimmune type reaction can be induced following infection [106–108]. Since AMD shares several features with autoimmune diseases, it could be proposed that infections could be at the origin of AMD. Historically the eye is known as an immune privilege site [109] protected against deleterious immune responses. However, it is now clear that the eye is able to elicit an immune response as needed, for instance, to respond to a viral infection [110]. Following elimination of the pathogen from the eye, an autospecific immunity may arise that will destroy ocular tissues [111, 112]. Various mechanisms exist including molecular mimicry, bystander activation, epitope spreading, and dual reactive B and T cells, which all could explain how a specific antibacterial or viral response can turn against RPE and/or retinal tissues [113–115]. Molecular mimicry, the most studied of these mechanisms, has been proposed to explain occurrence of many autoimmune diseases including multiple sclerosis, rheumatoid arthritis, or systemic lupus erythematous [113, 114]. Molecular mimicry may also occur in ocular tissues during an infection by a pathogen that contains antigens closely related to self-antigens present in the retina and/or the RPE and induce AMD. Indeed, it has been shown in susceptible strains of mice that following ocular infection with MHV viral particles are rapidly cleared from the eye; however, after 14 weeks, retinal degeneration occurs that is associated with the presence of autoantibodies specific of retinal and RPE antigens [116]. So far however, associations of AMD with infections by a pathogen have been scarce and inconsistent. In one study, AMD has been associated with elevated serum IgG titers specific to* Chlamydia pneumoniae* [117]. However, this has been refuted by Miller et al. [118]. In contrast, these same authors showed association of wet AMD with IgG titers against cytomegalovirus (CMV) [118]. Because of the small samples size in these studies and because environmental and host factors such as HLA and CFH polymorphisms are also involved in AMD, it will be difficult to definitely link one pathogen to AMD. Alternatively, autoimmune-like responses observed during AMD could be induced by infections with several pathogens that the host encounters during its entire life. This concept is called “total pathogen burden.” The hypothesis that prior infections with multiple pathogens carry more risk than infection with a single pathogen has been proposed to explain elevated levels of CRP during coronary artery disease [119]. Accordingly, taken together the sum of plasma levels of IgG against CMV,* Chlamydia pneumoniae* and* Helicobacter pylori*, correlates with the wet form of AMD [118]. Therefore the notion of “total pathogen burden” may prove useful in the future to better understand the pathology of AMD and may provide new biomarkers and therapeutic strategies against this disease.

## 6. Concluding Remarks

In summary, in addition to inflammation that is clearly involved in AMD pathogenesis [8, 10, 11] we have seen that adaptive immunity is also probably at play in this disease. Autoantibodies present in the sera of AMD patients, specifically those against PS and CEP adducts, and T cells (either producing IL-17 or not) could play a role in the pathogenesis of AMD and retinal aging by modifying the metabolic equilibrium of the retina and RPE or directly destroying retinal and RPE cells expressing autoantigens.

Concerning autoantibodies, despite the statistically significant associations of their presence during ARM (i.e., the very early stages of AMD), CNV, and GA [43, 44], their role during AMD remains a subject of debate [45]. More studies are needed to determine their exact role(s) during disease evolution.

Data reported herein clearly suggest that elevated levels of IL-17 in the sera and ocular tissues of AMD patients are involved in AMD pathogenesis. The knowledge that AMD is probably due to an autoimmune response mediated at least partly by IL-17 producing T cells may provide new therapeutic targets for the treatment of this disease [34]. For instance, Th-17^+^ T cells can be recruited to the CNS across the blood-retina barrier via several chemokine/chemokine receptor axes. IL-17 producing T cells have been reported to express mainly CCR2 [120], CCR6 [121], and CXCR3 [122]. Targeting any of these chemokine receptors during the course of AMD may prove beneficial for patients. Humanized-anti-IL-17 antibodies, secukinumab and ixekizumab, are now in phase II trial for the treatment of rheumatoid arthritis [123]. Results of these trials should prove useful regarding novel strategies for AMD therapeutic approaches. In addition, detection of specific autoantibodies and/or T cells related molecules may serve as early biomarkers of AMD allowing treatments to start before irreversible ocular tissues damage occurs.

The possible implications of oxidative stress and/or infection by inducing an autoimmune type reaction may also open new perspectives in understanding and treatment of AMD. The beneficial effects of antioxidant strategies observed during AMD [124] could therefore be explained not only by reduction of direct tissue damage induced by free radicals but also by reduced production of neoantigens leading to activation of the immune system against ocular tissues.

Altogether, evidence presented here suggests that autoimmunity is one of the main risk factors in AMD. Therefore in the future instead of age-related macular degeneration, the three letters A, M, and D may stand for autoimmune macular disease.

## Note

While this paper was in press, Cruz-Guilloty et al. [125] published the results of a study strongly suggesting that antigen-specific T cells play a leading role in AMD pathogenesis. These authors demonstrate CEP-specific T cells presence in the eyes of CEP immunized mice* in vivo*. These observations are in agreement with the hypothesis that DAMPs elicit T cells recruitment and activation in response to oxidative stress of the retina.
